# Extracellular Vesicles Secreted by TDO2-Augmented Fibroblasts Regulate Pro-inflammatory Response in Macrophages

**DOI:** 10.3389/fcell.2021.733354

**Published:** 2021-10-22

**Authors:** Kiel A. Peck, Alessandra Ciullo, Liang Li, Chang Li, Ashley Morris, Eduardo Marbán, Ahmed Gamal Ibrahim

**Affiliations:** Smidt Heart Institute, Cedars-Sinai Medical Center, Los Angeles, CA, United States

**Keywords:** extracellular vesicles, exosomes, inflammation, immunoregulation, macrophages, fibroblasts, TDO2

## Abstract

Extracellular vesicles (EVs) are secreted lipid bilayer vesicles that mediate cell to cell communication and are effectors of cell therapy. Previous work has shown that canonical Wnt signaling is necessary for cell and EV therapeutic potency. Tryptophan 2,3-dioxygenase (TDO2) is a target gene of canonical Wnt signaling. Augmenting TDO2 in therapeutically inert fibroblasts endows their EVs with immunomodulatory capacity including attenuating inflammatory signaling in macrophages. Transcriptomic analysis showed that macrophages treated with EVs from fibroblasts overexpressing TDO2 had blunted inflammatory response compared to control fibroblast EVs. *In vivo*, EVs from TDO2-overexpressing fibroblasts preserved cardiac function. Taken together, these results describe the role of a major canonical Wnt-target gene (TDO2) in driving the therapeutic potency of cells and their EVs.

## Introduction

Extracellular vesicles (EVs) are nano-sized lipid-bilayer vesicles secreted by nearly all cell types and represent an evolutionarily conserved mechanism of cell–cell communication (Yáñez-Mó et al., 2015; Zaborowski et al., 2015; Mathieu et al., 2019). EVs are broadly classified by their biogenesis (Meldolesi, 2018). Exosomes are smaller (30–100 nm) (Raposo and Stoorvogel, 2013) EVs that arise from the late endosome processed by the endosomal sorting complexes required for transport (ESCRT) pathway. Ectosomes which include microvesicles and apoptotic bodies, in contrast, are passively shed from the plasma membrane (ectosomes) (Mathieu et al., 2019; Sahoo et al., 2021). EVs are laden with potent signaling molecules including lipids, proteins, and RNA (Yáñez-Mó et al., 2015; Zaborowski et al., 2015; Mathieu et al., 2019). EV signaling plays a critical role in development, health, and disease (Ibrahim and Marban, 2016; Malloci et al., 2019). Emerging evidence also implicates EV secretion and signaling in the therapeutic effect of cell therapy (Ibrahim and Marban, 2016; Marban, 2018; Yin et al., 2020). Cardiosphere-derived cells (CDCs) are a population of cardiac stromal progenitors with demonstrated therapeutic bioactivity in cardiac and skeletal muscle indications. Early studies implicated CDC-EVs as mediators of the CDC therapeutic effect (Ibrahim et al., 2014; Gallet et al., 2017; Rogers et al., 2019). CDCs, through CDC-EVs, modulate several pathways of tissue healing and repair (Ibrahim et al., 2014), most notably, immunomodulation (de Couto et al., 2017, 2019). Furthermore, we and others identified macrophages as major functional recipients of CDC-EVs and mediators of therapy (de Couto et al., 2017, 2019). Macrophages are pivotal players in tissue injury and resolution. Recent mechanistic investigation by our group further implicated Wnt-β-catenin signaling activation as necessary for the secretion of therapeutic EVs by CDCs (Ibrahim et al., 2019, 2021). However, the specific downstream target genes of β-catenin signaling, and their effect on EV-cargo, remain poorly described. Here, we identify a β-catenin-upregulated target gene tryptophan 2,3-dioxygenase (TDO2). TDO2 is an enzyme involved in the metabolism of tryptophan into various metabolites including kynurenine with well-described roles in immunomodulation. Here we investigate the role of TDO2 activation in modulating macrophage inflammatory activation.

## Materials and Methods

### Neonatal Human Dermal Fibroblasts

Neonatal human dermal fibroblasts (nHDFs) were sourced from ATCC (PCS-201-010). Cells were cultured in IMDM (GIBCO), 10% FBS (Hyclone), 2 mM L-glutamine (GIBCO), and gentamicin (GIBCO). Cells were maintained at 37°C 20% O_2_/5% CO_2_ in complete medium with medium exchanges every 3–4 days as needed. Cells were grown until near confluent and passaged using TrypLE (GIBCO).

### Lentiviral Transduction

Neonatal human dermal fibroblasts were plated in T25 flasks and transduced with TDO2 activation lentiviral particles (Santa Cruz Biotech) at MOI:20 in complete medium. After 24 h of transduction, the virus was removed, and fresh complete medium was added for cell recovery for a further 24 h. Cells were then subjected to selection by 5.0 μg/mL puromycin for approximately 3–4 days. Following selection, complete medium was replaced and cells were grown and passaged.

### EV Preparation and Isolation

Extracellular vesicles were harvested from primary nHDFs at passage 5-7, from normal and transduced cells using a 15-day serum starvation method previously described (Walravens et al., 2021). Briefly, cells were grown to near confluence (∼90%) at 20% O_2_/5% CO_2_ at 37°C. Cell bed was washed 2x with warmed phosphate-buffered saline (PBS) and then incubated in IMDM without serum supplementation for 15 days in the same environment. Conditioned medium was collected, centrifuged at 3,000 × *g* for 10 min to remove dead cells and debris, then filtered through a 0.45-μm PES filter to remove apoptotic bodies and protein aggregates, and frozen for later use at −80°C. EVs were purified using centrifugal ultrafiltration with a 100-kDa molecular weight cutoff filter (Sigma-Millipore). EV preparations, before and after concentration were analyzed by NTA using the Malvern Nanosight NS300 Instrument (Malvern Instruments) with the following acquisition parameters: camera levels of 15, detection level less than or equal to 5, number of videos taken = 5, and video length of 30 s.

### Size-Exclusion Chromatography

Extracellular vesicles were collected and prepared as described above. After 100 kDa ultrafiltration, EVs were further purified using size exclusion chromatography (SEC) columns (SBI). Briefly, 1.0 mL of concentrated EVs was added to each chromatographic column and incubated at room temperature with rotation for 30–35 min. EVs were eluted from the column by centrifugation at 500 × *g*. EV size and concentration were analyzed by NTA as described above. Protein content of EV preparations was quantified using a BCA assay (Pierce).

### Bone Marrow-Derived Macrophages

Bone marrow-derived progenitor cells were collected from 3-month-old female Wistar Kyoto rats and differentiated into bone marrow-derived macrophages (BMDMs) by culturing with 20 ng/mL recombinant M-CSF (Life Technologies). Briefly, whole bone marrow cells were collected via aspiration with ice-cold PBS. Cells were filtered using a 70-μm cell strainer and centrifuged at 400 × *g* for 10 min at 4°C to pellet. The cell pellet was resuspended in 10 mL ACK buffer (GIBCO) for 30 s. ACK was quenched with IMDM + 10% FBS, and cells were centrifuged as described above. Cells were resuspended in complete medium; IMDM + 10% FBS + 20 ng/mL M-CSF and counted. Cells were seeded into six-well plates at 8.0e10^4^ cells/well, or equivalent. Cells were incubated at 37°C with 20% O_2_ and 5% CO_2_. Fresh complete medium was exchanged on day 3 and cells monitored for confluence. Test compounds were administered once BMDM cultures reached ∼75% confluence. Serum concentration was reduced to 1% during assays to facilitate EV uptake.

### Bromodeoxyuridine (BrdU) Assay

Primary bone marrow macrophages were collected as previously described and plated in 96-well plates at a density of 4.0e^4^ cells/well in complete medium (IMDM + 10% FBS + human recombinant M-CSF 20 ng/mL). After attachment and maturation (∼3 days), complete medium was removed and replaced with IMDM w/1% FBS for all test conditions. 4.0e^6^ EVs were added ∼1 h after medium change along with lipopolysaccharide (LPS) (10 ng/mL, Sigma). Control conditions were cultured in IMDM w/10% and 1% FBS. Cells were grown overnight after EV & LPS addition and proliferation quantified using a bromodeoxyuridine (BrdU) Cell Proliferation ELISA (Abcam, ab126556).

### Cell Migration (Modified Boyden Chamber Assay)

Bone marrow-derived monocytes were seeded onto 100 mm cell culture dishes and differentiated into mature macrophages using IMDM + 10% FBS + 20 ng/mL human recombinant M-CSF (Life Technologies). Upon reaching 75% confluence, BMDMs were lifted using ice-cold PBS + 2 mM EDTA. Cells were quantified and re-seeded onto 8.0-μM pore size transwells (Costar). Cells were recovered overnight at 37°C, 5% CO_2_ with complete medium (IMDM + 10% FBS). One hour before EV administration, complete medium was removed, cells were gently washed 1x with serum-free IMDM, and medium was replaced with IMDM + 1% FBS. Cells were incubated with EVs overnight and then fixed with 4.0% PFA. Cells were gently removed from the upper side of the transwell using a cotton swab. The underside of the transwell was stained for 20 min at RT using Crystal Violet. After staining, cells were gently washed several times with PBS until the wash ran clear. Ten images were captured of each transwell at 10x magnification (three per condition). Quantification of cell migration was done using ImageJ.

### RNA Isolation and RT-qPCR

Total cell RNA was isolated using the RNeasy Plus Mini Kit (Qiagen) according to the manufacturer’s protocol. Total EV RNA was isolated using the miRNeasy Advanced Serum Plasma Kit (Qiagen). Total cell RNA was quantified using NanoDrop and diluted using diH_2_O. Total EV RNA was quantified by Qubit (Thermo Fisher Scientific). Cellular RNA Reverse Transcription was performed using the High-Capacity RNA-to-cDNA kit (Life Technologies) with 1 μg RNA per reaction. PCR reactions were performed on the QuantStudio 7 Flex Real-Time PCR System (Applied Biosystems) using TaqMan Fast Advanced Master Mix (Life Technologies, 4444556) and TaqMan primers. Each reaction was performed in triplicate. The gene expression assays used for this study are summarized in Supplementary Table 1.

### Cell Lysate and Protein Assay

Cell lysates were collected for ELISA and western blot from six-well plates. Cells were washed 1x with ice-cold PBS. Cells were lysed in-well with 75 μL 1 × lysis buffer with phospo/protease inhibitors (Thermo Fisher Scientific). The cell lysate was incubated on ice for 15 min, sonicated twice for 10 s each, and centrifuged at 15,000 × *g* for 15 min at 4°C. The supernatant was collected and frozen for later use at −80°C. Protein lysates were quantified using a Pierce BCA Protein Assay kit (Thermo Fisher Scientific).

### Electrophoresis and Western Blot

Electrophoresis was conducted using NuPage 4–12% Bis-Tris protein gels (Life Technologies) using 25 μg protein per well. HPVD Membrane transfer was performed using the Turbo Transfer System (BIO-RAD) after gel electrophoresis. Blocking was performed using 5% non-fat milk in TBS + 20% Teen, 1 h at RT. Primary antibody staining was done overnight at 4°C. Secondary HRP antibody staining was done for 90 min at RT and then detected by SuperSignal West Pico PLUS Chemiluminescent Substrate (Thermo Fisher Scientific). Antibodies used in this study are summarized in Supplementary Table 2.

### ELISA

Interleukin-6 ELISA (R&D Systems, Quantikine ELISA) was performed according to the manufacturer’s protocol. Samples concentration for testing was 1.5 mg mL^–1^.

### RNA and miRNA Sequencing

Cell and EV RNA samples were sequenced at the Cedars-Sinai Genomics Core. Total RNA and Small RNA were analyzed using an Illumina NextSeq 500 platform for cell and EV samples, respectively.

### Proteomic Analysis of Extracellular Vesicles

Proteomics of nHDF-EV and nHDF^TDO2^-EV was conducted by Creative Proteomics (Shirley, NY, United States) using 200 μg protein per sample. Data analysis and processing were done using FunRich (Pathan et al., 2015).

### Animal Study

All animal studies were conducted under approved protocols from the Institutional Animal Care and Use Committee protocols.

### Mouse Acute Myocardial Infarction Model

Acute myocardial infarction was induced in 3-month-old male C57/B-L6 mice as described previously (Ibrahim et al., 2014). Within 10 min of left anterior descending artery ligation, a total of 1 × 10^5^ cells (or vehicle) were administered via 3 × 8μL injections intramyocardially.

### Echocardiography

Echocardiography was performed in the mouse model of acute myocardial infarction at 1 day (baseline) and 21 days after surgery using Vevo 3100 Imaging System (Visual Sonics) as described (Ibrahim et al., 2019). The average of the left ventricular ejection fraction was analyzed from multiple left ventricular end-diastolic and left ventricular end-systolic measurements.

### Statistics

GraphPad Prism 9.0 (GraphPad Software) was used to analyze the data. A comparison of three or more groups was performed using two-way or one-way ANOVA followed by Sidek’s *post hoc* multiple comparison test for paired groups. Two-group comparisons were analyzed using two-tailed unpaired *t*-tests with a confidence interval of 95%. RNA sequencing data were analyzed for differential expression, fold change, and unsupervised PCA using DESeq2 (Anders and Huber, 2010; Love et al., 2014).

## Results

### TDO2 Augmentation Results in Broad Gene Expression Changes in Fibroblasts

Normal human dermal fibroblasts (HDFs) treated with the beta-catenin activator 6-bromoindirubin-3’-oxime (BIO) lead to upregulation of TDO2 more than any other gene (Supplementary Figure 1A). Therefore, to examine the role of TDO2, we transduced neonatal human fibroblasts (nHDFs) with lentivirus containing the TDO2 transgene under the control of a constitutive promoter. Upon transduction and subsequent selection by puromycin, expression of TDO2 was 50-fold higher than non-transduced cells (Supplementary Figure 1B). TDO2 activation had a significant impact on the nHDF transcriptome. Transcriptomic sequencing identified 3,000 differentially expressed genes and over 400 unique genes in TDO2 transduced cells (Supplementary Figures 1D–E). Increased expression of TDO2 was further confirmed by the sequencing data as well (Supplementary Figure 1F).

### TDO2-Expressing Fibroblasts Attenuate Macrophage Activation

**FIGURE 1:**
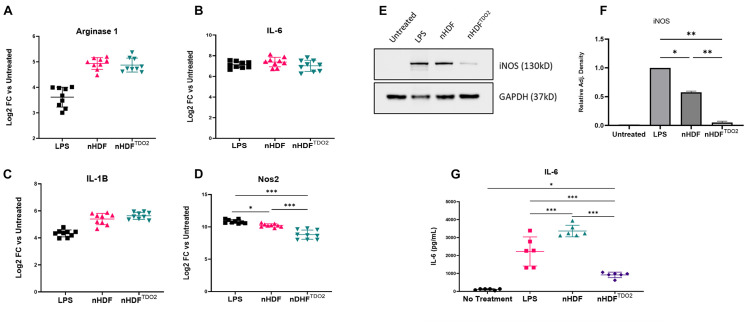
Transwell co-culture of bone marrow-derived macrophages. **(A–D)** Gene expression of inflammatory genes after co-culturing of cells with nHDF or nHDF^TDO2^ cells (*n* = 3 triplicates from three independent experiments). **(E)** Representative image of western blot detection of iNOS in BMDM lysates after overnight co-culture with nHDF or nHDF^TDO2^ cells and pooled data (*n* = 2 biological replicates). **(F)** Analysis was done using one-way ANOVA with Sidak’s multiple comparison test. Error bars represent standard deviation. ^∗^*p* < 0.05, ^∗∗^*p* < 0.01, and ^∗∗∗^*p* < 0.001.

To explore the effect of TDO2 activation on the immunomodulatory capacity of nHDFs, rat BMDMs were co-cultured with TDO2-transduced nHDFs (nHDF^TDO2^), un-transduced nHDFs (nHDF^UNT^), or LPS as an activation control. Examination of macrophage polarization and inflammatory markers showed no changes in arginase 1, IL-6, and IL-1B expression following co-culture (Figures 1A–C). Nos2 expression was significantly decreased compared to both LPS- and nHDF-co-cultured BMDMs (Figure 1D). Decreased expression of the protein, iNOS, was further confirmed by western blot (Figures 1E,F). Interestingly, while no changes were observed in IL-6 transcription (Figure 1B), secreted IL-6 in the conditioned culture medium was lower as observed by ELISA which suggests potential post-translational regulation (Figure 1G). To investigate the potential mediator of the immunoregulatory effect of TDO2-activated nHDFs, we assessed the levels of secreted kynurenine. TDO2 is a rate-limiting enzyme in the conversion of L-tryptophan to *N*-formyl-L-kynurenine. Kynurenine and its downstream metabolites play roles in anti-inflammation and vascular relaxation (Nguyen et al., 2010; Wang et al., 2010). However, subsequent analysis of conditioned medium by ELISA revealed no increase in kynurenine (Supplementary Figure 1C). Therefore, TDO2 activation endows nHDFs with immunomodulatory capacities as shown by their ability to blunt IL-6 and Nos2 expression in co-cultured macrophages. Furthermore, this effect is not driven by the synthesis of kynurenine.

### TDO2-Augmented Fibroblasts EVs Are Enriched in Small Non-coding RNA

**FIGURE 2:**
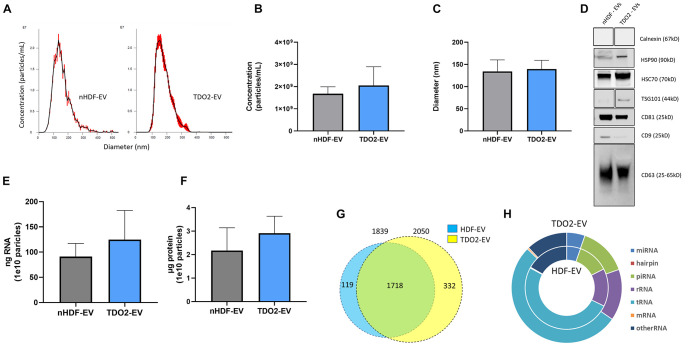
nHDF and nHDF^TDO2^ derived extracellular vesicles share a similar phenotype. **(A)** Nanosight NS300 analysis of 15-day, serum-starved, conditioned medium, from nHDF and nHDF^TDO2^ cultures (*n* = 3 technical replicates). **(B,C)** Particle concentration and particle diameter as observed by NTA (*n* = 3 triplicates from three independent experiments). **(D)** Western blot of common EV markers. **(E)** Total RNA was extracted from 1.0e^10^ EVs and quantified using Qubit (*n* = 4 from two biological replicates). **(F)** EV protein content was analyzed by micro-BCA from 1.0e^10^ particles per sample (*n* = 4 from two biological replicates). **(G)** miRNA sequencing aligned 2,169 miRNA sequences with annotated miRNA. **(H)** Ratio of RNA species found during sequencing. Sequencing data were derived from three biological replicates from each nHDF and nHDF^TDO2^ derived EV.

Having ruled out the role of kynurenine, we investigated changes in EV payload post TDO2 activation in nHDFs. nHDF^TDO2^ (nHDF^TDO2^-EVs) and control nHDF EVs (nHDF-EVs) were conditioned using a 15-day serum-starvation protocol described by us previously (Ibrahim et al., 2014). EVs were isolated using ultrafiltration with 100 kDa molecular weight cut-off followed by buffer exchange with PBS. The size and concentration of EVs were similar between the nHDF and nHDF^TDO2^ groups (Figures 2A–C). Further characterization was completed demonstrating the presence of conserved EV markers such as HSP70, CD81, and CD63 (Figure 2D). The absence of the endoplasmic reticulum (ER) protein Calnexin in both preparations demonstrates equivalent purification during EV concentration (Figure 2D). Total RNA and protein content were comparable in both groups (Figures 2E,F). Subsequent RNA sequencing of the vesicles revealed a slight enrichment of total small RNAs in the TDO2 group (Figure 2G). This increase appeared to be primarily driven by increases in piRNA and tRNA (Figure 2H).

### EVs From TDO2-Augmented Fibroblasts Attenuate Macrophage Activation

**FIGURE 3:**
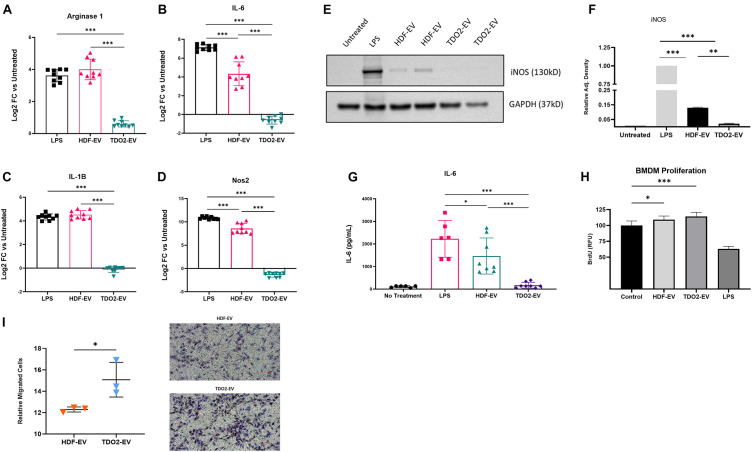
nHDF^TDO2^-EVs show anti-inflammatory function in bone marrow-derived macrophages. **(A–D)** Gene expression of inflammatory genes in BMDM cells after overnight treatment with nHDF or nHDF^TDO2^ EVs results graphed as log2 fold change versus untreated cells (not shown) (*n* = 3 triplicates from three independent experiments). **(E)** Representative image of western blot detection of iNOS in BMDM cell lysates (*n* = 2 from biological replicates). **(F)** Quantification of iNOS western blot images. **(G)** ELISA quantification of IL-6 in the cell culture conditioned medium of BMDMs treated overnight with nHDF-EV or nHDF^TDO2^-EVs (*n* = 3 triplicates from three independent experiments). **(H)** Proliferation of BMDM cells analyzed by colorimetric BrdU incorporation assay (*n* = 8 technical replicates from a single experiment). **(I)** Results of modified Bowden-chamber assay for cell migration (*n* = 3, 10 technical replicates; images from three independent experiments). **(A–H)** Analysis was done using one-way ANOVA with Sidek’s multiple comparisons test. **(I)** Analysis was done using an unpaired, two-tailed, *t*-test. All error bars represent standard deviation. ^∗^*p* < 0.05, ^∗∗^*p* < 0.01, and ^∗∗∗^*p* < 0.001.

To investigate the function of EVs from nHDF^TDO2^, BMDMs were treated directly with EVs from each group and assayed for the same markers of macrophage and inflammatory markers described earlier. Significant reductions were observed in the expression of Arg-1, Nos2, IL-1B, and Nos2, and ADAM17 was observed after overnight treatment with EVs (Figures 3A–D and Supplementary Figure 4A). Additional inflammation markers were investigated but were not observed to have significantly changed expression levels (Supplementary Figure 4B). Reduced iNOS levels in BMDM were confirmed by western blot (Figures 3E,F). Reductions in IL-6 secretion were observed by ELISA (Figure 3G). Both EV groups also stimulated BMDM proliferation equally as shown by BrdU incorporation (Figure 3H). The effect of EVs on migration was also evaluated using a modified Bowden’s Chamber assay. Interestingly, nHDF^TDO2^-EVs enhanced macrophage migration compared to other groups (Figure 3I). Taken together, these data demonstrate a potent immunomodulatory effect of nHDF^TDO2^ in macrophages. This effect was reflected by inherent changes in the inflammatory profile of the cells rather than impairing proliferation or infiltration. To rule out bioactivity from extra-vesicle proteins, EV preparations were further purified using SEC. SEC purification yielded preparations with nearly 10-fold less protein compared to the ultrafiltration only preparation (Supplementary Figure 2A). Particle numbers were equivalent between both groups (Supplementary Figures 2B,C). Equivalently to the ultrafiltration product, SEC-purified EVs attenuated Nos2 and IL6 (Supplementary Figures 2E,F) which suggests that the immunomodulatory effects on macrophages are mediated by the EVs. Additionally, very few differences were observed when comparing the proteomic composition of nHDF and nHDF^TDO2^ derived EVs (Supplementary Figure 3). These data suggest that EV-associated proteins are not responsible for the immunomodulatory effects observed.

### EVs From TDO2-Augmented Fibroblasts Induce Macrophage “Anergy” via Suppression of NFkB

**FIGURE 4:**
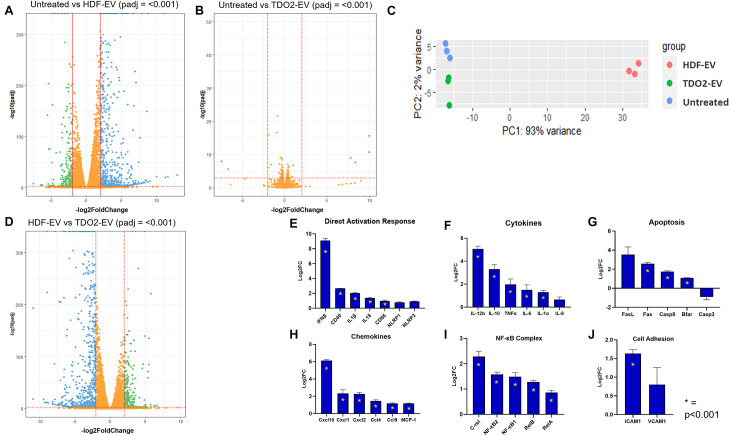
RNA sequencing of EV-treated bone marrow-derived macrophages (BMDMs) reveals activation of inflammatory pathways by nHDF-EV. Differential expression analysis was calculated from *n=3* from two biological replicates. **(A)** Volcano plot visualizing the changes in gene expression in BMDM cells after treatment with nHDF-EVs compared to the untreated group. **(B)** Volcano plot visualizing the changes in gene expression in BMDM cells after treatment with nHDF^*TDO2*^-EVs compared to the untreated group. **(C)** Principal component plot of the top 300 differentially expressed genes (*p* < 0.001). **(D)** Volcano plot visualizing the changes in gene expression in BMDM cells after treatment with nHDF^*TDO2*^-EVs compared to the nHDF-EV treated group. All volcano plots use −log10 adjusted *p*-values and log2 fold-changes. Genes with adjusted *p*-values less than 0.001 and fold-changes greater than twofold are highlighted. **(E–J)** Log2 fold change gene expression changes in inflammatory markers when BMDMs are treated with nHDF-EV vs nHDF^*TDO2*^-EV. Log2FC values calculated from FPKM count values, *n=3*, error bars represent standard deviation, and starred plots indicate *p* < 0.001.

To investigate the mechanism by which EVs from nHDF^TDO2^ regulate macrophage activation, we performed transcriptomic analysis on macrophages treated with nHDF^TDO2^-EVs and nHDF-EVs. Macrophages treated with nHDF-EVs had significant transcriptomic changes including the activation of several pro-inflammatory genes (Figure 4A). In contrast, macrophages treated with nHDF^TDO2^-EVs had a much more muted effect (Figure 4B). This is further reflected using principle component analysis where nHDF^TDO2^-EV-treated groups are more proximal to the untreated group than the nHDF-EV-treated group (Figure 4C). Indeed, nHDF^TDO2^-EVs nearly reverse the inflammatory effects of nHDF-EV treatment (Figure 4D). Given this major reversal of inflammatory phenotype, we interrogated nuclear factor-kappa b (NFκB) genes as it is a central regulator of inflammation (Taniguchi and Karin, 2018). Analysis of the NFκB inflammatory complex shows potent abatement compared to the nHDF-EV-treated group and equivalent to the untreated group (Supplementary Figure 4G). Direct comparison of canonical inflammatory markers and pathways shows universal upregulation in macrophages treated with nHDF-EVs versus those treated with nHDF^TDO2^-EVs (Figures 4E–J) including decreased expression of all constituents of the NFκB complex (Figure 4I). This led us to suspect whether the nHDF^TDO2^-EVs might be inert and incapable of signaling to macrophages. To test this hypothesis, we performed a sequential exposure experiment whereby macrophages were treated first with nHDF^TDO2^-EVs followed by exposure to nHDF-EVs. If the nHDF^TDO2^-EVs are truly inert, then the nHDF-EVs should still induce inflammatory activation. If the nHDF^TDO2^-EVs induce anergic modulation in macrophages, then macrophage activation by nHDF-EVs will be attenuated. Indeed, pre-treating macrophages with nHDF^TDO2^-EVs blunted their ability toward inflammatory activation by nHDF-EVs. This is shown by decreased levels of Arg-1, Nos2, and IL-6, and a significant increase in IL-10 expression after treatment with nHDF-EVs (Supplementary Figures 4C–F). Taken together, these data demonstrate a unique ability of nHDF^TDO2^-EVs to induce immunomodulation through inducing anergia in macrophages. This effect is mediated in part through blunting NFκB activation.

### TDO2-Augmented Fibroblasts Are Cardioprotective in Acute Myocardial Infarction

**FIGURE 5:**
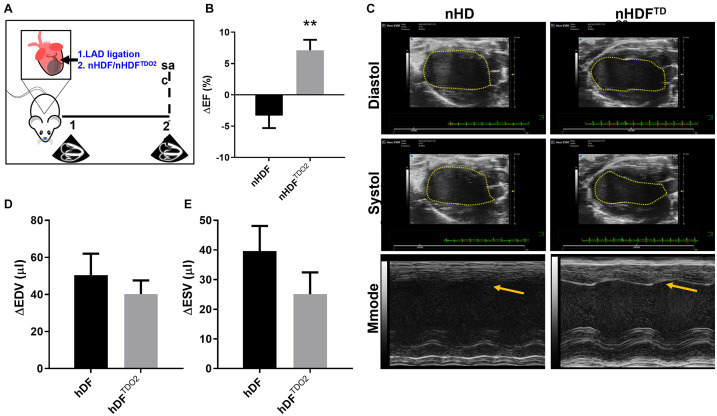
Neonatal human fibroblasts transduced to overexpress TDO2 increase ejection fraction in a 3-week mouse model of MI. **(A)** In an acute model of MI, BL6 mice underwent MI and hearts were injected with nHDF cells (*n* = 10) and nHDF^TDO2^ cells (*n* = 8). **(B)** Treatment with nHDF^TDO2^ cells increased ejection fraction in mice 21-day post-infarct. **(C)** Representative B-mode images of systole and diastole illustrating the ventricular-volume tracing method used to measure ejection fraction. M-mode images of left ventricular contraction. Improved contraction in the left ventricular wall after treatment with nHDF^TDO2^ cells is observed by the inward contraction of the left ventricular wall (yellow arrow). **(D)** Average change in left ventricular end-diastolic volumes. **(E)** Average change in left ventricular end-systolic volume. Statistical analysis was done using an unpaired *t*-test. **p < 0.01.

Having observed these profound effects in macrophages, we sought to investigate the effect of nHDF^TDO2^ in a cardiac injury model where macrophages play an active role in injury and resolution. We investigated the therapeutic capacity of these cells in a well-established mouse model of acute myocardial infarction used by our group to establish therapeutic potency of cells and EVs (Figure 5A). Results showed significant improvement of cardiac function at 21-day post-injury in nHDF^TDO2^-treated hearts (compared to nHDF-treated hearts; Figure 5B). Observation of both B-mode M-mode images reveals an observable difference in left ventricular wall contractility (Figure 5C). Changes in end-diastolic and systolic volumes were decreased in the nHDF-TDO2-EV treated hearts, indicating preservation of end-systolic volumes and maintenance of ejection fraction (Figures 5D,E).

## Discussion

Understanding the mechanism of action of cell therapy is a cornerstone of regenerative medicine. Previous work by us and others suggests that cell therapy functions primarily through the secretion of EVs (Barile et al., 2014; Ibrahim et al., 2014; Hirai et al., 2020) which deliver molecules like small RNAs with salutary effects that modulate the transcriptome of the injured microenvironment, notably macrophages (de Couto et al., 2017, 2019). The further mechanistic investigation implicated the Wnt-β catenin pathway in driving the therapeutic effect of CDCs and their EVs (Ibrahim et al., 2014, 2021). The work presented here represents a continuation of this mechanistic dissection. We show that TDO2 is a major target gene of β catenin activation. Indeed, it was the single highest upregulated gene in fibroblasts with augmented β catenin activation. TDO2 activation in otherwise therapeutically negative cells (skin fibroblasts) resulted in a secretome capable of regulating macrophage inflammatory activation. This effect was independent of kynurenine synthesis and secretion but instead was mediated by changes in the cargo of EVs. EVs isolated from the conditioned medium of TDO2-augmented cells were potently immunomodulatory in macrophages. Deeper transcriptomic analysis of macrophages treated with nHDF^TDO2^-EVs revealed potent silencing of pro-inflammatory properties of nHDF EVs including activation of a master regulator of inflammatory signaling NFκB. This effect was not due to rendering EVs non-reactive but rather an active anti-inflammatory signaling process. Finally, we show that fibroblasts augmented with TDO2 were therapeutically bioactive in a model of cardiac injury. In conclusion, these results shed light on one target gene (TDO2) of β catenin activation and its role in the therapeutic effect. Future work will focus on changes in the cargo of EVs upon TDO2 activation that endow them with immunomodulatory capacity. For instance, it will be important to interrogate changes in the non-coding RNA including micro RNAs (miRs) and their immune-relevant targets. This includes members of the NFκB pathway. This investigation would not be confined to miRs but rather other RNA classes with less described mechanisms that may contribute significantly to the observed effects. Of note are the Piwi-RNAs (piRNAs) which increased in EVs following TDO2 activation. Furthermore, the effect of these EVs on the adaptive immune response was not evaluated here and merits investigation as TDO2 activation has been shown to induce immunological tolerance. Understanding the comprehensive effect of EVs from TDO2-activated cells further advances our growing knowledge of therapeutic signaling by cells and the EVs and more broadly, the relevant mechanisms in tissue healing and repair.

## Data Availability Statement

The datasets presented in this study can be found in online repositories. The names of the repository/repositories and accession number(s) can be found below: ExoCarta database under the accession number: ExoCarta_313.

## Ethics Statement

The animal study was reviewed and approved by the Institutional Animal Care and Use Committee, Cedars-Sinai Medical Center.

## Author Contributions

KP was the primary contributor, conducted all *in vitro* experiments and bioinformatics, authored the first draft of the manuscript, created all figures, and performed all statistical testing. LL contributed animal surgery support and captured echocardiographs. AC and CL contributed to the method development and study design. AM provided analytical support. EM and AI provided conceptual study design and project oversight. All authors contributed to the article and approved the submitted version.

## Conflict of Interest

EM owns founder stock in Capricor Therapeutics. The remaining authors declare that the research was conducted in the absence of any commercial or financial relationships that could be construed as a potential conflict of interest.

## Publisher’s Note

All claims expressed in this article are solely those of the authors and do not necessarily represent those of their affiliated organizations, or those of the publisher, the editors and the reviewers. Any product that may be evaluated in this article, or claim that may be made by its manufacturer, is not guaranteed or endorsed by the publisher.

## Supplementary Material

Supplementary Figure 1 Augmented expression of TDO2 has a global effect on the gene expression profile of neonatal human fibroblasts. **(A)** Sequencing of cardiosphere-derived cells (CDCs) treated with BIO revealed TDO2 to be the gene most upregulated. **(B)** TDO2 expression 2 passages after lentiviral transduction and selection by puromycin. **(C)** Secreted kynurenine levels from nHDF versus nHDF^TDO2^ cell cultures as tested by ELISA (*n* = 5). **(D)** Visualization of mRNA sequencing data showing global gene expression in nHDFs before and after transduction. Including significantly (*p* ≤ 0.001) differentially expressed genes (direction delineated by colored arrows). **(E)** Heat map visualizing differentially expressed genes (*p* < 0.001) post TDO2 augmentation. **(F)** Volcano plot visualizing the expression of TDO2 in the group of genes upregulated post-transduction. Unpaired, two-tailed *t*-test used for analysis. Error bars represent standard deviation. ^∗^*p* < 0.05, ^∗∗^*p* < 0.01, and ^∗∗∗^*p* < 0.001.

Supplementary Figure 2 Removal of extra-vesicle protein does not change inhibit immunomodulatory function in BMDM. **(A)** Total protein concentration of ultrafiltration and SEC purified EV preparations. **(B)** EV concentrations normalized by volume. **(C)** EVs particle per μg of protein. **(D–F)** Arg1, Nos2, and IL-6 expression in BMDMs treated with different preparations of TDO2 and HDF EVs. Two-group comparisons were analyzed using an unpaired, two-tailed *t*-test. Multiple comparisons were tested by one-way ANOVA with Sidek’s multiple comparison test. Error bars indicate standard deviation. ^∗^*p* < 0.05, ^∗∗^*p* < 0.01, and ^∗∗∗^*p* < 0.001.

Supplementary Figure 3 Proteomic analysis of nHDF and nHDF^TDO2^ derived extracellular vesicles. **(A)** Visualization of the ratio of differentially expressed proteins (576) and the number of proteins mapped from each EV type, including unique sequences from nHDF (32) and nHDF^TDO2^ (13). **(B)** Comparison of the ratio of proteins based on their biological processes and molecular function. **(C)** Proteins significantly up- or downregulated in the TDO2-EV population delineated by their biological processes.

Supplementary Figure 4 Pre-treatment with nDHF^TDO2^-EVs reduces the inflammatory reaction in BMDM. **(A)** Gene expression of ADAM17 in macrophages after treatment with EVs. **(B)** Expression of genes indicating M1 and M2 polarization. Fold change calculated versus untreated cells. **(C–F)** Gene expression of inflammatory markers after standard treatment with EVs or nHDF^TDO2^-EV (*n* = 3). **(G)** Heat map illustrating the gene expression of the NFκB inflammatory complex and TLR adapter proteins MyD88 and TRIF after EV treatment versus controls (*n* = 3 triplicates from two independent experiments). Two-group comparisons were analyzed using an unpaired, two-tailed *t*-test. Multiple comparisons were tested by one-way ANOVA with Sidek’s multiple comparison test. Error bars indicate standard deviation. ^∗^*p* < 0.05, ^∗∗^*p* < 0.01, and ^∗∗∗^*p* < 0.001.
