# Supplementary figures and images for: Extracellular Vesicles Secreted by TDO2-Augmented Fibroblasts Regulate Pro-inflammatory Response in Macrophages
Source: Front Cell Dev Biol. 2021 Oct 22;9:733354. doi: 10.3389/fcell.2021.733354 (PMC8571098; doi:10.3389/fcell.2021.733354)

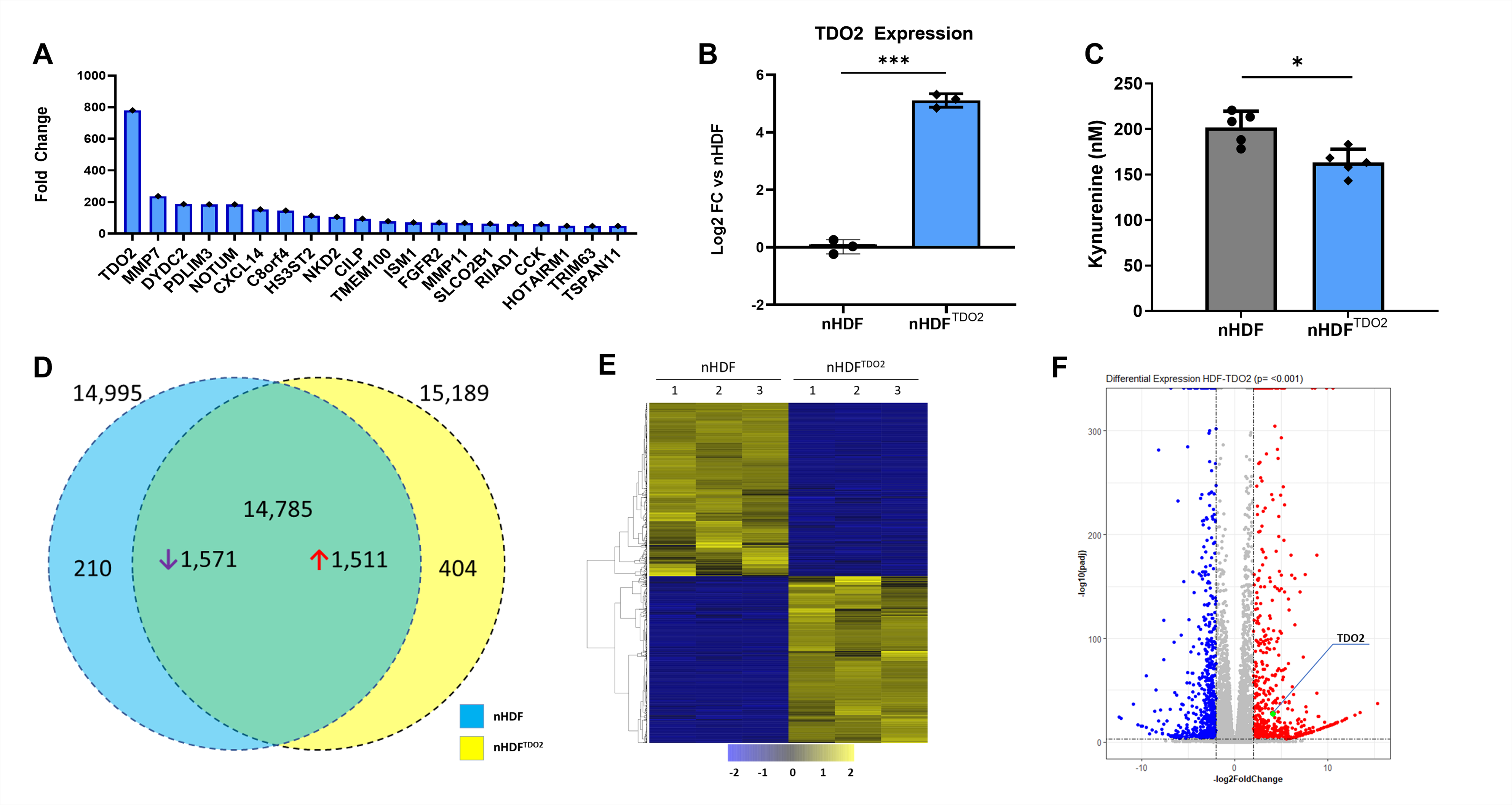

Supplement: Supplementary Figure 1 — Augmented expression of TDO2 has a global effect on the gene expression profile of neonatal human fibroblasts. (A) Sequencing of cardiosphere-derived cells (CDCs) treated with BIO revealed TDO2 to be the gene most upregulated. (B) TDO2 expression 2 passages after lentiviral transduction and selection by puromycin. (C) Secreted kynurenine levels from nHDF versus nHDFTDO2 cell cultures as tested by ELISA (n = 5). (D) Visualization of mRNA sequencing data showing global gene expression in nHDFs before and after transduction. Including significantly (p ≤ 0.001) differentially expressed genes (direction delineated by colored arrows). (E) Heat map visualizing differentially expressed genes (p < 0.001) post TDO2 augmentation. (F) Volcano plot visualizing the expression of TDO2 in the group of genes upregulated post-transduction. Unpaired, two-tailed t-test used for analysis. Error bars represent standard deviation. ∗p < 0.05, ∗∗p < 0.01, and ∗∗∗p < 0.001. [file Image_1.tif]

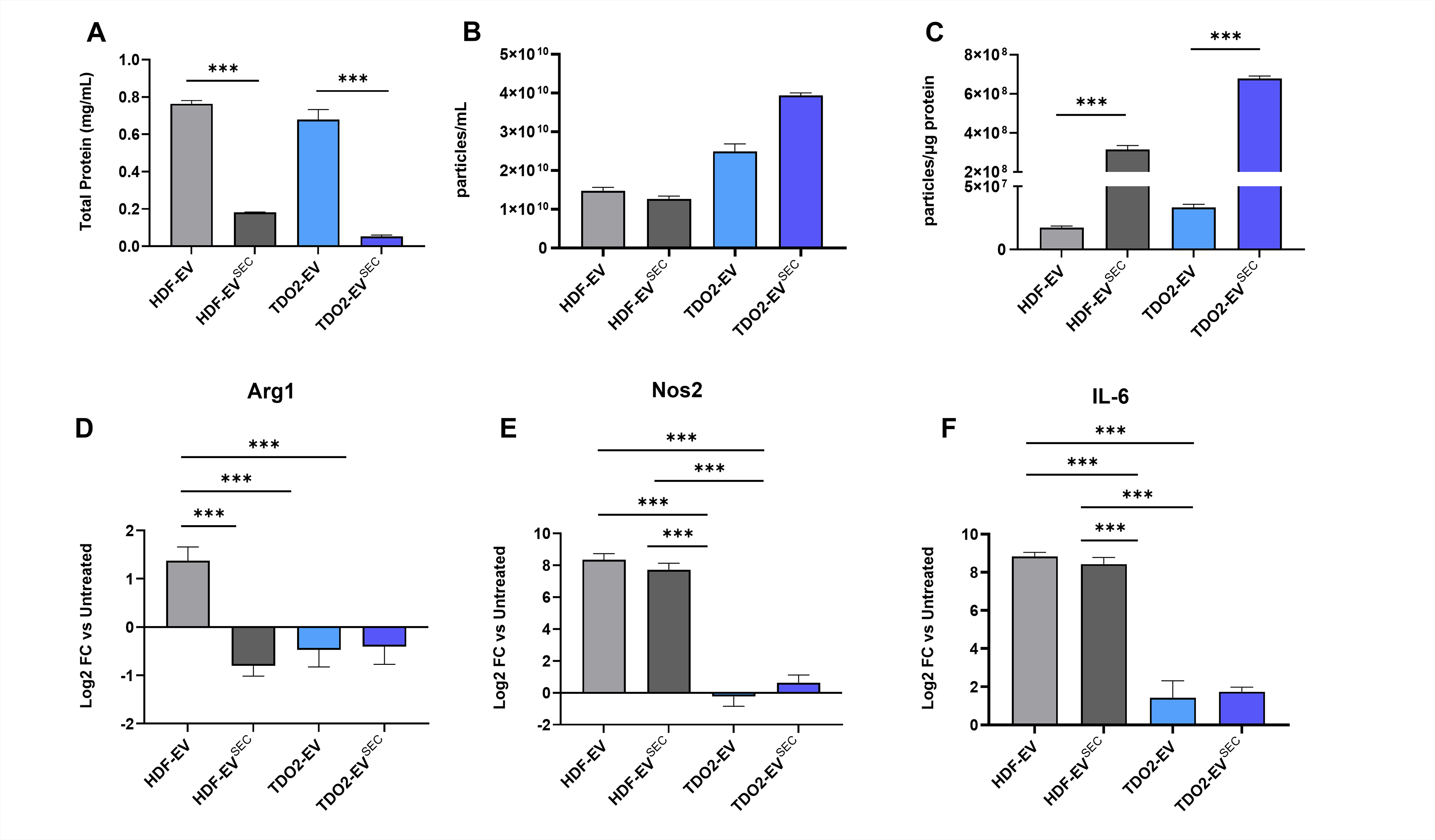

Supplement: Supplementary Figure 2 — Removal of extra-vesicle protein does not change inhibit immunomodulatory function in BMDM. (A) Total protein concentration of ultrafiltration and SEC purified EV preparations. (B) EV concentrations normalized by volume. (C) EVs particle per μg of protein. (D–F) Arg1, Nos2, and IL-6 expression in BMDMs treated with different preparations of TDO2 and HDF EVs. Two-group comparisons were analyzed using an unpaired, two-tailed t-test. Multiple comparisons were tested by one-way ANOVA with Sidek’s multiple comparison test. Error bars indicate standard deviation. ∗p < 0.05, ∗∗p < 0.01, and ∗∗∗p < 0.001. [file Image_2.TIF]

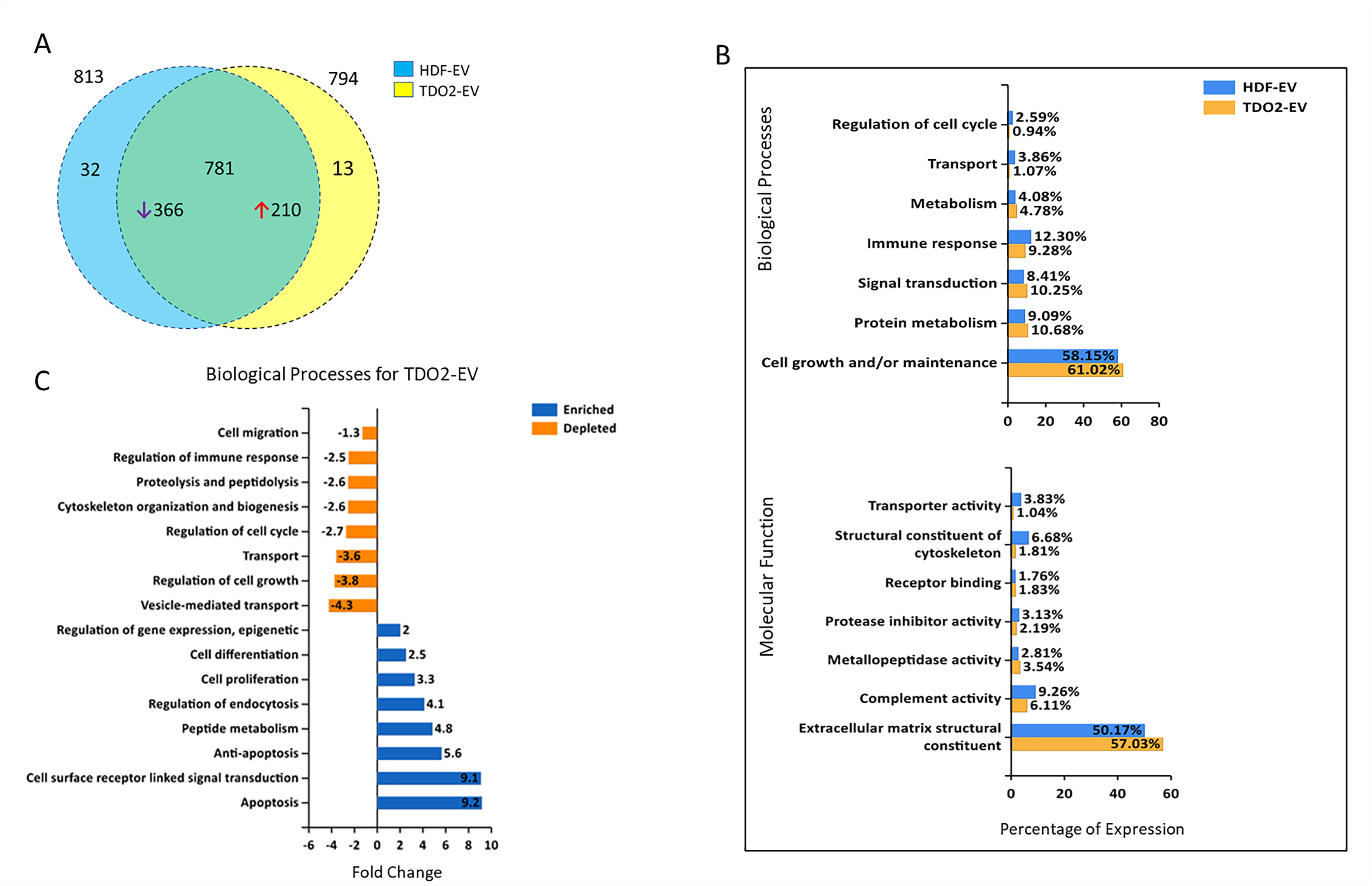

Supplement: Supplementary Figure 3 — Proteomic analysis of nHDF and nHDFTDO2 derived extracellular vesicles. (A) Visualization of the ratio of differentially expressed proteins (576) and the number of proteins mapped from each EV type, including unique sequences from nHDF (32) and nHDFTDO2 (13). (B) Comparison of the ratio of proteins based on their biological processes and molecular function. (C) Proteins significantly up- or downregulated in the TDO2-EV population delineated by their biological processes. [file Image_3.TIF]

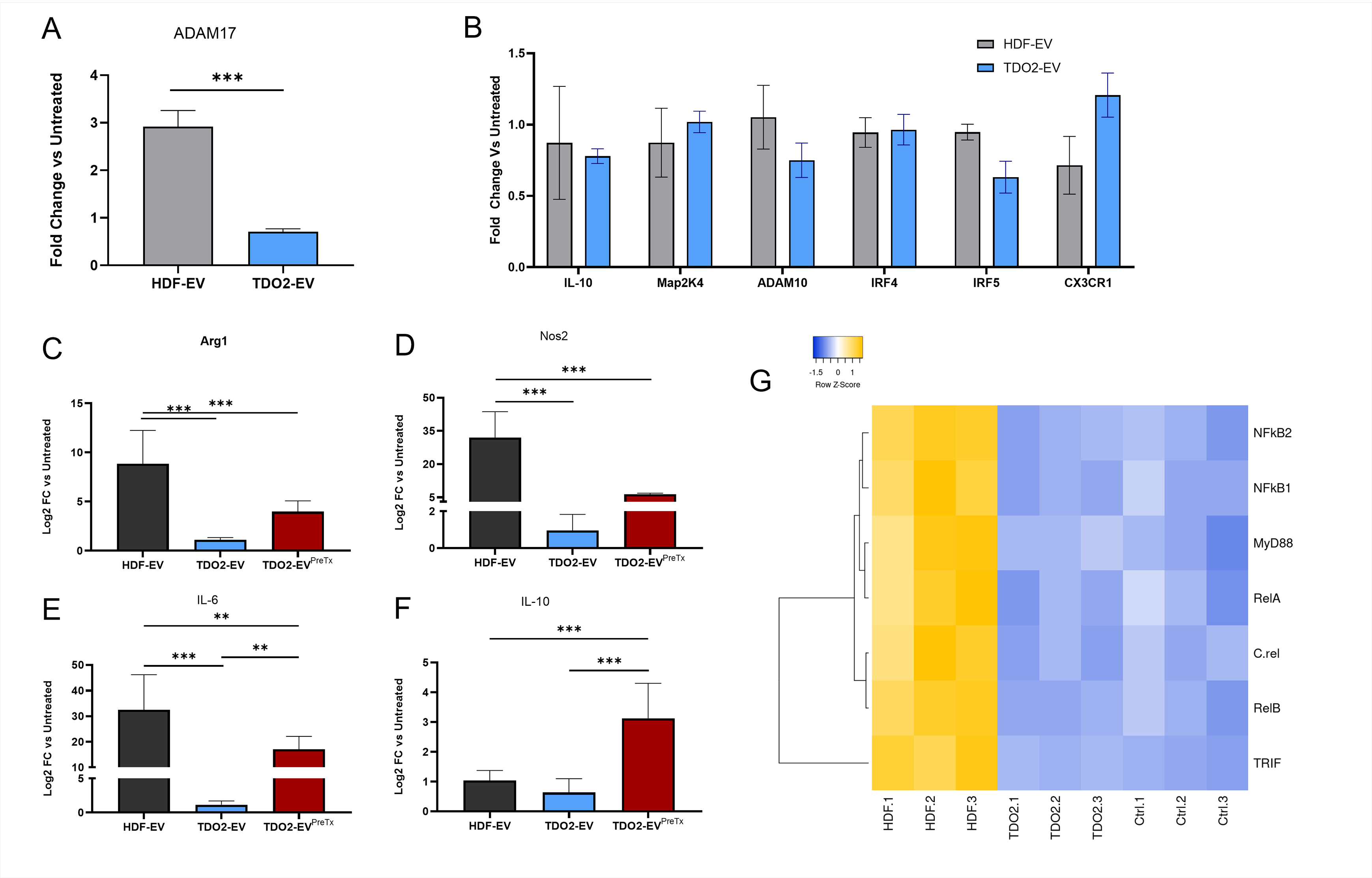

Supplement: Supplementary Figure 4 — Pre-treatment with nDHFTDO2-EVs reduces the inflammatory reaction in BMDM. (A) Gene expression of ADAM17 in macrophages after treatment with EVs. (B) Expression of genes indicating M1 and M2 polarization. Fold change calculated versus untreated cells. (C–F) Gene expression of inflammatory markers after standard treatment with EVs or nHDFTDO2-EV (n = 3). (G) Heat map illustrating the gene expression of the NFκB inflammatory complex and TLR adapter proteins MyD88 and TRIF after EV treatment versus controls (n = 3 triplicates from two independent experiments). Two-group comparisons were analyzed using an unpaired, two-tailed t-test. Multiple comparisons were tested by one-way ANOVA with Sidek’s multiple comparison test. Error bars indicate standard deviation. ∗p < 0.05, ∗∗p < 0.01, and ∗∗∗p < 0.001. [file Image_4.TIF]
