# Supplementary material for: Extracellular Vesicles Secreted by TDO2-Augmented Fibroblasts Regulate Pro-inflammatory Response in Macrophages
Source: Front Cell Dev Biol. 2021 Oct 22;9:733354. doi: 10.3389/fcell.2021.733354 (PMC8571098; doi:10.3389/fcell.2021.733354)
Supplement: Supplementary file 5 [file Table_1.pdf]

**Supplementary Table 1: Gene Expression Assays**		
**Assay Names**	**Species**	**Assay ID**
TDO2	Human	Hs00355049_m1
HPRT1	Rat	Rn01527840_m1
Arg1	Rat	Rn00691090_m1
IL-6	Rat	Rn01410330_m1
Nos2	Rat	Rn00561646_m1
IL-10	Rat	Rn01483988_g1
IL-1b	Rat	Rn00580432_m1
Map2K4	Rat	Rn01461524_m1
ADAM10	Rat	Rn01530753_m1
ADAM17	Rat	Rn00571880_m1
Cx2cr1	Rat	Rn00591798_m1
IRF4	Rat	Rn01435145_m1
IRF5	Rat	Rn01500522_m1

**Supplementary Table 2: Antibodies**			
**Antibody Names**	Primary/Secondary	Company	Catalog Numbers
GAPDH (14C10) Rabbit mAb-HRP Conjugated	Primary	Cell Signaling Technology	3683
GAPDH (GA1R) Mouse mAb-HRP Conjugated	Primary	Invitrogen	MA5-15738
iNOS pAB Rabbit-IgG	Primary	Novus Biologicals	NB300-605
Calreticulin (D3E6) XP mAB Rabbit-IgG	Primary	Cell Signaling Technology	12238
HSP70 (3A3) mAB Mouse-IgG	Primary	Invitrogen	MA3-006
HSP90	Primary	Abcam	Ab13492
CD9 mAB Mouse IgG	Primary	BD Biosciences	555370
CD63	Primary	Invitrogen	106280
CD81 mAB Mouse IgG	Primary	BD Biosciences	555675
Anti-Rabbit IgG, HRP-Linked Antibody	Secondary	Cell Signaling Technology	7074
Anti-Mouse IgG, HRP-Linked Antibody	Secondary	Cell Signaling Technology	7076
